# Scandium Microalloying for Al-Cu-Mg-Mn-Ti Alloys with High Cu Content

**DOI:** 10.3390/ma19143095

**Published:** 2026-07-18

**Authors:** Junbao Guo, Hao Dong, Yuan Wang, Yuqian Wang, Zhenjie Zhao, Ruihong Zhang, Zekang Li, Yanfeng Liang, Zhong Yang

**Affiliations:** 1 School of Materials and Chemical Engineering, Xi’an Technological University, Xi’an 710021, China; junb_guo@163.com (J.G.); 18729322915@163.com (R.Z.); liangyan1979@163.com (Y.L.); 2 Shanxi Diesel Engine Industry Co. Ltd., Datong 037001, China; 3 Engineering Training Center, Xi’an Technological University, Xi’an 710021, China; 15029000040@163.com

**Keywords:** Al-Cu alloy, Sc element, grain refinement, microstructure, mechanical property

## Abstract

Increasing the Cu content is an important approach to enhancing the strength of Al-Cu alloys, but the widening of the solidification temperature range leads to deterioration in the casting performance of the alloy, significantly limiting its application. This work investigates the effect of Sc content on the microstructure and mechanical properties of Al-Cu-Mg-Mn-Ti alloys with high Cu content. The results show that the solidification path of the Al-8.5Cu-0.3Mg-0.35Mn-0.2Ti-xSc alloy changes when the Sc content reaches 0.37%. When the Sc content is less than 0.37%, the primary phase is α-Al, whereas when the Sc content is greater than 0.37%, the primary phase is Al3Sc. As the Sc content increases to 0.4%, the grain size of the alloy first decreases and then increases, with the smallest grain size occurring at a Sc addition of 0.3 wt.%. Accordingly, the tensile strength of the alloy at both room temperature and high temperature reaches the highest when the Sc content is 0.3 wt.%. Specifically, the alloy’s tensile strength at room temperature reaches 327.2 MPa, an increase of 14.0% compared to the alloy without Sc, and the alloy’s tensile strength reaches 103 MPa at 350 °C, an increase of 24.5% compared to the alloy without Sc. It is speculated that when Sc is added to the Al-Cu melt, it refines the grains by forming Al3Sc heterogeneous nucleation cores, and it may also form Sc-containing precipitates with higher temperature resistance, which boosts the alloy’s strength.

## 1. Introduction

The Al-Cu alloys have the advantages of low density, high specific strength, and easy machinability, and are widely used in parts in transportation and aerospace, such as airplane skins, engine pistons, truck wheels, and airplane load-bearing frames [1,2,3,4]. Generally the increase in strength of Al-Cu alloys depends on the increase in the Cu content. Higher Cu content allows for the precipitation of a higher density of age-hardening phase Al2Cu, thereby enhancing the alloy’s strength [5,6,7,8]. However, at the same time, as the Cu content increases, the alloy’s solidification temperature range expands rapidly, resulting in a wider “mushy zone” during solidification [9,10]. This causes the alloy’s fluidity and shrinkage compensation ability to decrease sharply, leading to hot cracking at grain boundaries in Al-Cu alloys with high Cu content [11,12,13,14,15]. This seriously affects the forming quality and mechanical properties of castings made from such materials.

Adding microalloying elements is an important method for controlling the forming quality and mechanical properties of Al-Cu alloys. Among them, the Sc element, due to its high melting point (1541 °C) and small diffusion coefficient in the aluminum matrix [16,17,18], easily forms high-thermal-stability nanoscale Sc-containing precipitates or enriches at the interfaces of inherent precipitates such as Al2Cu to stabilize phases like Al2Cu [19,20,21,22,23], significantly enhancing the alloy’s temperature resistance, and has received increasing attention in recent years. For example, Xue et al. [24] demonstrate that adding Sc to Al-Cu-Mg-Ag alloys causes it to diffuse into the lattice of the aging precipitate θ-Al2Cu and form a Sc-containing V phase. The V phase maintains a large volume fraction while exhibiting higher thermal stability, enabling the Al-Cu-Mg-Ag-Sc alloy to achieve a tensile strength of up to 100 MPa at 400 °C. The addition of Sc elements leads to the transformation of the age-hardening precipitate θ-Al2Cu into the more temperature-resistant V phase, which was also confirmed by Coello et al. [25]. Yang et al. [26] show that Sc-vacancy clusters can effectively inhibit solute diffusion in cast Al-Cu-Li alloys, thereby delaying the precipitation of copper-containing phases and suppressing the continuous coarsening of the δ′ phase, which in turn significantly enhances the mechanical properties of the alloy. Yang et al. [27] indicate that in Al-Cu-Sc alloys, Sc atoms tend to segregate at the interface between θ′ and the α-Al matrix. This interfacial solute segregation not only promotes the nucleation of the θ′ precipitate phase but also significantly inhibits its coarsening, resulting in a greatly increased density of θ′ precipitates in the alloy and, consequently, a substantial enhancement of the alloy’s strength. Allami et al. [28] studied the effect of Sc microalloying on the solidification structure and mechanical properties of AZ91 alloy. The results showed that with the addition of Sc, the alloy formed thermally stable Al3Sc and Mg5Al4Sc particles, which significantly enhanced the creep performance of the alloy over a wide range of temperatures and stress levels. This indicates that adding the microalloying element Sc is highly effective in regulating the solidification structure of aluminum alloys and improving their mechanical properties.

It should be emphasized that in order to effectively reduce hot cracking that occurs during the solidification of such alloys, thereby preventing the deterioration of the mechanical properties, most existing studies on Sc microalloying have focused on Al-Cu alloys with relatively low Cu content (e.g., 4.5–5.5 wt.% [24,25,26,27,28]), and have paid more attention to the effects of Sc on the alloy’s mechanical properties at room and high temperatures. However, the addition of Sc can form an Al3Sc phase with the same crystal structure and a similar lattice constant as the primary α-Al phase, and the heterogeneous nucleation of the Al3Sc phase can significantly refine the grain size of the primary α-Al phase [19,20,21,22,23,29,30]. This can improve the filling and shrinkage compensation properties of Al-Cu alloys, thereby effectively suppressing the occurrence of hot cracking. Therefore, this work aims to study the effect of adding different mass fractions of Sc to high-copper Al-Cu alloys on their microstructure and mechanical properties, laying a foundation for improving the heat resistance and broader industrial applications of these materials.

## 2. Methodology

The raw materials used in this work are commercial pure Al (99.95 wt.%) and pure Mg (99.90 wt.%), Al-50Cu (wt.%), Al-10Ni (wt.%), Al-5Ti (wt.%), and Al-2Sc (wt.%) master alloys. The chemical compositions of the alloys with different Sc contents used in this work are listed in Table 1. Before the alloy melting, dry the prepared materials in an oven at 150 °C. Then, melt the pure aluminum below 720 °C in a medium-frequency electromagnetic induction furnace. Once it melts, raise the temperature to 800 °C and sequentially add the Al-50Cu (wt.%), Al-10Ni (wt.%), Al-5Ti (wt.%), and Al-2Sc (wt.%) intermediate alloys and hold for 10 min to ensure the master alloys are completely melted. Then cool the melt to 730 °C, and add pure Mg and melt it. Finally, cast the molten metal at 730 °C into a steel mold preheated to 200 °C, then allow it to cool naturally to room temperature.

The microstructure of the samples was observed using a JSM-7200F scanning electron microscope (SEM) (Japan Electronics Co., Ltd., Tokyo, Japan) equipped with an Energy-Dispersive Spectrometer (EDS) and a JEM-2010 transmission electron microscope (TEM) (Japan Electronics Co., Ltd., Tokyo, Japan). Room-temperature tensile tests were carried out on a GMT6203 tensile testing machine (Sansi Eternal Technology Co., Ltd., Zhejiang, China) at a speed of 0.5 mm/min. High-temperature tensile tests were conducted using a D2-0200-1 electronic universal testing machine with a constant speed of 0.5 mm/min and a temperature of 350 °C. During the experiment, the sample was heated at a rate of 10 °C/min, and held at the target temperature for 15 min, and then the test was conducted. For the room-temperature and high-temperature tensile tests, three specimens of each alloy were tested. Thermodynamic calculations were performed using Pandat 2022 software to analyze the phase composition and solidification path of the alloy.

## 3. Results and Discussion

### 3.1. Microstructure of Al-8.5Cu-Mg-Mn-Ti-xSc Alloy

**Figure 1:**
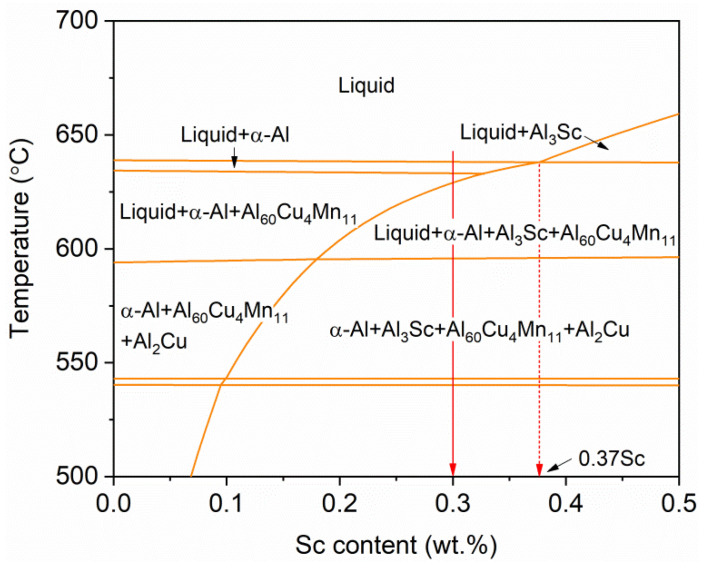
Equilibrium phase diagram of Al-8.5Cu-Mg-Mn-Ti-xSc alloy.

The equilibrium solidification phase diagram of Al-8.5Cu-0.3Mg-0.35Mn-0.2Ti-xSc alloys with different Sc additions is created using Pandat 2022 software. As shown in Figure 1, the solidification path of the alloy changes obviously when the Sc content is about 0.37 wt.%. That is, when the Sc content is less than 0.37 wt.%, the alloy undergoes the L→α-Al reaction to precipitate the primary α-Al phase, whereas when the Sc content is greater than 0.37 wt.%, the alloy undergoes the L→Al3Sc reaction to precipitate the primary Al3Sc phase. When the Sc content is exactly 0.37 wt.%, the alloy undergoes a eutectic reaction (i.e., L→α-Al + Al3Sc) to precipitate the α-Al and Al3Sc phases. Thereafter, as the temperature decreases, the residual melt solidifies phases such as Al2Cu.

**Figure 2:**
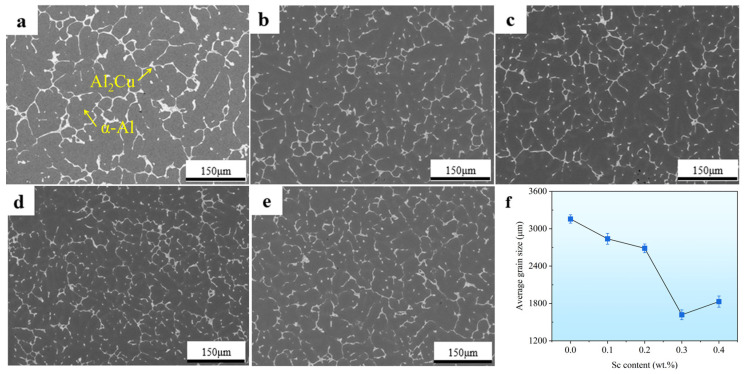
Microstructure of as-cast Al-8.5Cu-0.3Mg-0.35Mn-0.2Ti-xSc alloys: (**a**) 0 wt.% Sc; (**b**) 0.1 wt.% Sc; (**c**) 0.2 wt.% Sc; (**d**) 0.3 wt.% Sc; (**e**) 0.4 wt.% Sc; (**f**) The average grain size varies with the content of Sc.

The microstructure of as-cast Al-8.5Cu-0.3Mg-0.35Mn-0.2Ti-xSc alloys with different Sc additions was analyzed using an SEM, as shown in Figure 2a–e. Figure 2f presents the grain size statistics of the microstructures in Figure 2a–e. It can be seen that the as-cast Al-8.5Cu-0.3Mg-0.35Mn-0.2Ti-xSc alloys are composed of the α-Al matrix and solidification phases at the grain boundaries (Figure 2a–e). With the increase in Sc content, the grain size of the alloys first decreases and then increases, with the most significant refinement observed at 0.3 wt.% Sc addition (Figure 2f). As shown in Figure 1, the alloy’s solidification path changes at 0.37% Sc. That is, when the Sc content is below this value, the primary phase of the alloy is α-Al, followed by the solidification of phases like Al_3_Sc. On the other hand, when the Sc content is above 0.37%, the primary phase is Al_3_Sc, followed by the solidification of α-Al and other phases. Therefore, when the current multicomponent alloy solidifies over a relatively wide temperature range (for the 0.3% Sc alloy, the phase diagram in Figure 1 shows that the alloy’s solidification temperature range is over 100 °C), the primary α-Al dendrites may break during the filling process, forming uniform nucleation particles. Additionally, during subsequent cooling, after the Al_3_Sc phase solidifies, they can act as a heterogeneous nucleation core for the following α-Al phase. In other words, the grain refinement of the 0.37% Sc alloy is due to both dendrite fragmentation and heterogeneous nucleation by the Al_3_Sc phase. For the 0.4% Sc alloy, the primary phase is Al_3_Sc. Since it is near the eutectic point (0.37% Sc), there is less primary Al_3_Sc forming during solidification, resulting in limited heterogeneous nucleation.

**Figure 3:**
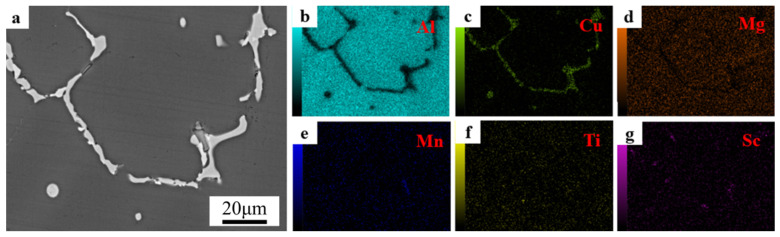
Surface scan of microstructure of as-cast Al-8.5Cu-Mg-Mn-Ti-0.3Sc alloy: (**a**) surface scan area; (**b**–**g**) element distribution.

**Figure 4:**
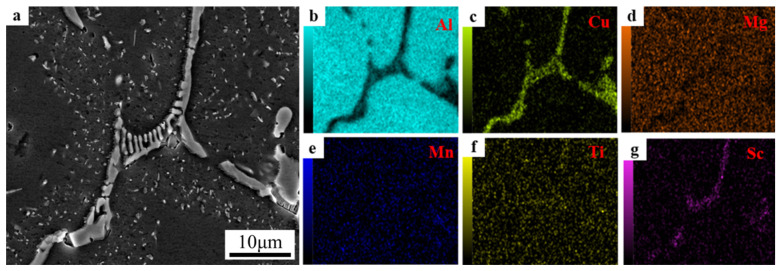
Surface scan of microstructure of Al-8.5Cu-Mg-Mn-Ti-0.3Sc alloy after T6 heat treatment: (**a**) surface scan area; (**b**–**g**) element distribution.

The distributions of elements in the Al-8.5Cu-0.3Mg-0.35Mn-0.2Ti-0.3Sc alloy after casting and T6 treatment are shown in Figure 3 and Figure 4, respectively. It can be seen from Figure 3 that the Mg, Mn, and Ti elements are all dispersed throughout the alloy matrix (Figure 3d–f). In contrast, besides being dispersed in the matrix, the Cu and Sc elements show significant segregation in the grain boundary regions (Figure 3c,g). Based on the equilibrium phase diagram of the Al-8.5Cu-0.3Mg-0.35Mn-0.2Ti-0.3Sc alloy shown in Figure 1, it can be seen that during the solidification of the alloy, the α-Al phase precipitates first. As the α-Al phase precipitates, the content of Cu and Sc in the melt gradually increases. As the melt temperature continues to decrease, the melt sequentially solidifies and precipitates the Al3Sc phase (L→Al3Sc; Figure 1) and the Al2Cu phase (L→α-Al + Al2Cu; Figure 1). During the eutectic reaction, the α-Al phase in the eutectic structure tends to nucleate on the primary α-Al phase, while the Al2Cu phase precipitates at the interface of the primary α-Al phase, exhibiting the typical features of a divorced eutectic structure (Figure 3a,c). After T6 heat treatment, as shown in Figure 4a, a high-volume fraction of aging precipitates forms in the alloy matrix. EDS analysis indicates that the aging precipitates in the matrix are mainly Al2Cu phases. In addition, typical high-Sc-content aging precipitates form at the alloy grain boundaries (Figure 4a,g). From Figure 4c,g, it can be seen that the distribution of Sc and Cu overlaps somewhat in the grain boundary regions. Whether this forms the V phase or W phase (Al8-xCu4xSc) [24,25] described in the literature, or some other Cu- and Sc-containing phase, still needs higher-resolution experimental characterization and confirmation.

**Figure 5:**
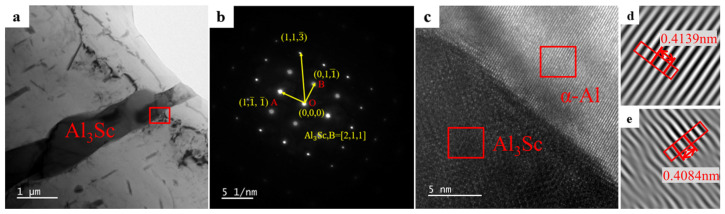
TEM microstructure of Al-8.5Cu-Mg-Mn-Ti-0.3Sc alloy after T6 heat treatment: (**a**) bright-field image; (**b**) electron diffraction result; (**c**) interface structure between α-Al and Al3Sc phases; measurement of interplanar spacing of (**d**) α-Al and (**e**) Al3Sc phases.

The morphology of the phases in the α-Al matrix of the Al-8.5Cu-0.3Mg-0.35Mn-0.2Ti-0.3Sc alloy after heat treatment was analyzed using TEM-based bright-field imaging (Figure 5a), electron diffraction (Figure 5b), and high-resolution characterization (Figure 5c,d). As can be seen from Figure 5a, aside from the finely dispersed Al2Cu precipitates in the alloy matrix, there are also coarse, elongated phases present. Through diffraction pattern calibration (calibration results: OA = 2.3711, OB = 1.4557, ∠AOB = 90.08; Figure 5b), these were identified as the Al3Sc phase. Figure 5c shows the high-resolution interface structure of the red-framed region in Figure 5a. Calculations indicate that the lattice mismatch between the Al3Sc phase and the α-Al matrix is about 1.3%, meaning the Al3Sc phase can serve as a heterogeneous nucleation site for the α-Al matrix. This also explains why, as the Sc content increases, grain refinement continuously occurs in the Al-8.5Cu-0.3Mg-0.35Mn-0.2Ti alloy, as observed in Figure 2.

### 3.2. Mechanical Properties of Al-8.5Cu-Mg-Mn-Ti-xSc Alloy

**Figure 6:**
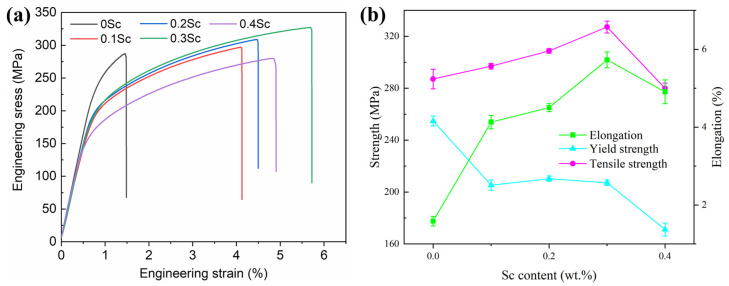
Room-temperature mechanical properties of Al-8.5Cu-0.3Mg-0.35Mn-0.2Ti-xSc alloy: (**a**) stress–strain curves; (**b**) corresponding values of tensile strength and elongation from the stress–strain curves in Figure 6a.

Tensile tests were carried out at room temperature on T6 heat-treated Al-8.5Cu-0.3Mg-0.35Mn-0.2Ti-xSc alloys, as shown in Figure 6a. Figure 6b presents the corresponding values of tensile strength and elongation from the tensile stress–strain curves in Figure 6a. As shown in the figures, under room-temperature conditions, as the Sc content increases from 0.1 wt.% to 0.4 wt.%, the tensile strength of the alloy first increases and then decreases, reaching its maximum at 0.3 wt.% Sc, where the alloy exhibits a tensile strength of 327.2 MPa. This represents a 14.0% increase compared to the alloy without Sc addition (i.e., Al-8.5Cu-0.3Mg-0.35Mn-0.2Ti alloy; 287.1 MPa). Surprisingly, the elongation at fracture of the alloy also first increases and then decreases as the Sc content increases from 0.1 wt.% to 0.4 wt.%, reaching its maximum at 0.3 wt.% Sc, where the elongation is 5.8%. This is 2.7 times higher than the alloy without Sc addition (i.e., 1.58%). Therefore, at 0.3 wt.% Sc, the alloy exhibits the optimal combination of tensile strength and elongation. The mechanism behind the changes in room-temperature tensile strength and fracture elongation with increasing Sc content will be discussed in detail in Section 3.3.

**Figure 7:**
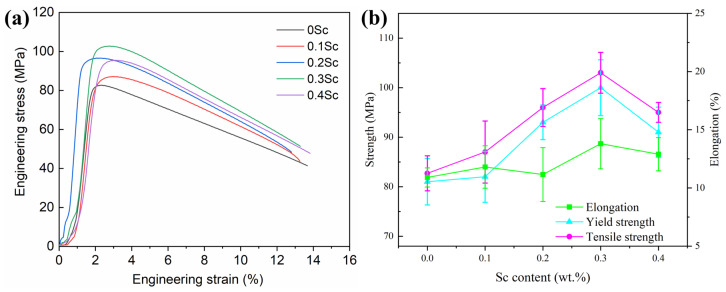
High-temperature (350 °C) mechanical properties of Al-8.5Cu-0.3Mg-0.35Mn-0.2Ti-xSc alloys: (**a**) stress–strain curves; (**b**) corresponding values of tensile strength and elongation from the stress–strain curves in Figure 7a.

Tensile tests were carried out at 350 °C on T6 heat-treated Al-8.5Cu-0.3Mg-0.35Mn-0.2Ti-xSc alloys, as shown in Figure 7a. Figure 7b shows the corresponding values of tensile strength and elongation extracted from the stress–strain curves in Figure 7a. As shown in the figures, when the alloy is stretched at high temperature (350 °C), its tensile strength first increases and then decreases with increasing Sc content. Among them, when the Sc content is 0.3 wt.%, the tensile strength of the alloy reaches the maximum value of 103 MPa. Compared with the Al-8.5Cu-0.3Mg-0.35Mn-0.2Ti alloy without Sc addition, the tensile strength is increased by 24.5%. In addition, as the Sc content increases from 0.1 wt.% to 0.4 wt.%, the alloy’s fracture elongation does not change significantly, fluctuating between 10% and 14% with increasing Sc content.

### 3.3. Fracture and Strengthening Mechanisms of Al-8.5Cu-Mg-Mn-Ti-xSc Alloy

**Figure 8:**
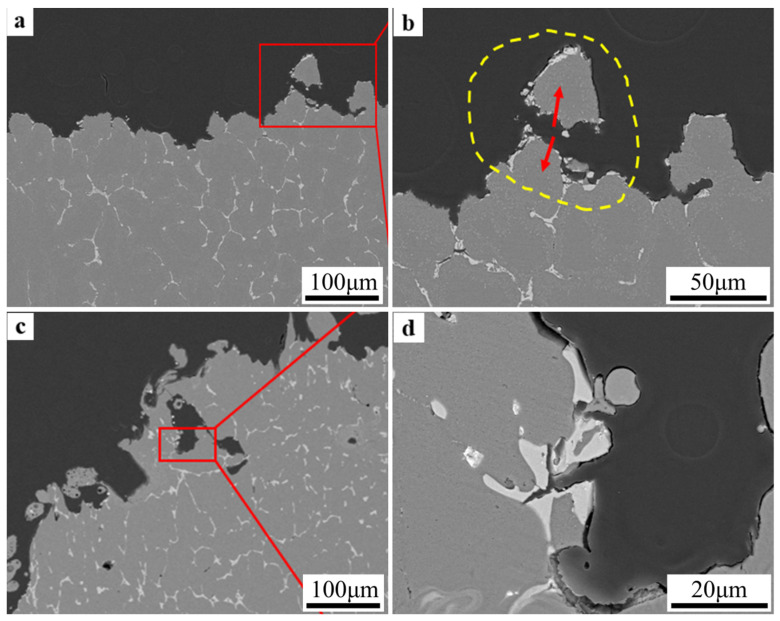
Fracture morphology of Al-8.5Cu-0.3Mg-0.35Mn-0.2Ti-0.3Sc alloy during room-temperature (**a**–**b**) and high-temperature (**c**–**d**) tensile tests.

The microstructures of the Al-8.5Cu-0.3Mg-0.35Mn-0.2Ti-0.3Sc alloy near tensile fracture at room temperature (Figure 8a,b) and high temperature (Figure 8c,d) are analyzed. As seen in Figure 8a,b, when the alloy is deformed at room temperature, the crack path propagates along the phase boundaries between Al2Cu and α-Al, displaying typical intergranular fracture characteristics. Generally, the deformation of aluminum alloys at room temperature is dominated by intragranular dislocation slip, so their fracture mode is usually transgranular [31,32,33,34]. It is discovered that the reason the current Al-8.5Cu-based alloy exhibits the abnormal intergranular fracture is that the alloy has a relatively wide solidification temperature range (about 90 °C), resulting in a significant amount of “mushy zones” during solidification. This causes the last-to-solidify regions between adjacent grains to often not feed properly, leading to microcracks at the grain boundaries due to solidification shrinkage, which become weak points in the alloy’s microstructure. During tensile deformation, the microcracks at the grain boundaries propagate, resulting in intergranular fracture of the alloy.

As can be seen from Figure 2 and Figure 5, with the addition of Sc and the increase in its content, the grain size of the primary α-Al phase gradually decreases due to the formation of Al3Sc heterogeneous nuclei. The reduction in the grain size of the primary α-Al phase can strengthen the alloy through grain refinement, following the Hall–Petch relationship:

*σ_y_* = *σ*_0_ + *kd*^−1/2^ (1)

where *d* is the grain size, and *σ*_0_ and *k* are material constants [34]. Based on the grain sizes shown in Figure 2f and the reported values of *σ*_0_ (60 MPa [35]) and *k* (0.21 MPa·m^−1/2^ [35]) from the literature, *σ_y_* can be calculated using Equation (1). The corresponding values are 0 wt.% Sc (63.75 MPa), 0.1 wt.% Sc (63.96 MPa), 0.2 wt.% Sc (64.2 MPa), 0.3 wt.% Sc (65.25 MPa), and 0.4 wt.% Sc (64.8 MPa). From the above calculations, it can be seen that as the Hall–Petch parameter *k* of aluminum alloy is relatively small (i.e., 0.21 MPa·m^−1/2^ [35]), the direct contribution of grain refinement to strength is not obvious. Based on the existing literature, adding Sc to Al-Cu alloys may lead to the formation of more temperature-resistant V or W phases [24,25]. As we know, aging strengthening is the main strengthening mechanism for Al-Cu alloys, generally accounting for more than 70% of the alloy’s strengthening effect [36]. So, it is inferred that the increase in alloy strength (Figure 6 and Figure 7) with higher Sc content is closely related to the formation of these V and W phases. In addition, for alloys with a high Cu content (i.e., Al-8.5Cu) compared to conventional Al-Cu alloys treated with solution and aging, where the Cu content is often below the maximum solubility of 5.65%, refining the grain size can significantly improve the alloy’s fluidity and shrinkage compensation ability, reducing the formation of developed primary dendrites that divide the melt into isolated pools and cause casting defects such as shrinkage cavities and porosity. Based on the above analysis, as the Sc content increases, the alloy grains are refined, the defects at grain boundaries are greatly reduced, and the bonding strength at the grain boundaries is significantly enhanced, which together lead to a substantial increase in the alloy’s room-temperature tensile strength. Additionally, due to grain refinement, plastic deformation is distributed among more grains, reducing local stress concentration and effectively delaying crack initiation, allowing the material to withstand greater plastic deformation. This also explains the observed phenomenon in Figure 6 where the alloy’s strength and elongation increase simultaneously.

As shown in Figure 8c,d, intergranular cracking also occurs in the alloy during high-temperature tensile deformation at 350 °C. On the one hand, as revealed by the room-temperature deformation mentioned above, the wide crystallization temperature range leads to reduced grain boundary cohesion. On the other hand, during high-temperature deformation of aluminum alloys, the deformation mechanism differs from that at room temperature [37,38]. Compared with room-temperature deformation, which is dominated by intragranular dislocation slip, when the temperature approaches or exceeds 350–400 °C, the deformation involves both intragranular dislocation slip and grain boundary sliding. That is, during high-temperature deformation, the grain boundary strength and stability of aluminum alloys are significantly reduced, becoming weak points prone to intergranular cracking [39,40]. Based on the above analysis, with the addition of Sc and the increase in its content, the grains of the Al-8.5Cu-0.3Mg-0.35Mn-0.2Ti-xSc alloy are refined, and the grain boundary bonding strength also increases, which together lead to an increase in the alloy’s strength at both room and high temperatures.

## 4. Conclusions

In this work, the effects of adding different mass fractions of scandium on the microstructure and mechanical properties of Al-Cu alloys were investigated. The main conclusions are summarized below:

(1) When the Sc content in the Al-8.5Cu-0.3Mg-0.35Mn-0.2Ti-xSc alloy is less than 0.37%, the primary phase α-Al solidifies; otherwise, the primary phase Al3Sc solidifies. When the Sc content is 0.37%, the alloy undergoes a eutectic reaction to precipitate the α-Al and Al3Sc eutectic phase.

(2) As the Sc content increases to 0.4%, the grain size of the alloy first decreases and then increases, with the grain refinement effect being most significant at a Sc addition of 0.3 wt.%; additionally, when the Sc content increases to 0.3 wt.%, after aging treatment, the Sc-containing precipitates form at the grain boundary area.

(3) The tensile strength of the Al-8.5Cu-0.3Mg-0.35Mn-0.2Ti-xSc alloy first increases and then decreases with the increase in Sc content. When the Sc content is 0.3 wt.%, it reaches a maximum of 327.2 MPa at room temperature and 103 MPa at 350 °C. Compared with the alloy without Sc, the tensile strength at room temperature and high temperature increased by 14.0% and 24.5%, respectively. It is inferred that grain refinement and the formation of high-temperature-resistant Sc-containing precipitates lead to the improvement of the alloy’s performance.

## Figures and Tables

**Table 1:** Chemical compositions of the Al-Cu-Mg-Mn-Ti-Sc alloy used in this work.

Elements	Cu	Mg	Mn	Ti	Sc	Al
Mass fraction (%)	8.5	0.3	0.35	0.2	0-0.4	Bal.

## Data Availability

The original contributions presented in this study are included in the article. Further inquiries can be directed to the corresponding authors.
